# Application of Bark Beetle Semiochemicals for Quarantine of Bark Beetles in China

**DOI:** 10.1673/031.006.4101

**Published:** 2006-11-16

**Authors:** Yong Liu, Huaguo Dai

**Affiliations:** College of Plant Protection, Nanjing Agricultural University, Nanjing, 210095, China

**Keywords:** Scolytidae, Platypodidae, Bostychidae and Cleridae, *Xyleborus*, *Cryphalus*, *Polygraphs*, *Xyloterus*, *Ips*, *Dendroctonus*, *Orthotomicus*, *Scolytus*

## Abstract

This article describes the use of bark beetle semiochemicals for quarantine in China. Using traps with two isomeric compounds of α-pinene, ethanol, trans-verbenol, verbenone, camphene and isononylaldehyde, insects of four families (Scolytidae, Platypodidae, Bostychidae and Cleridae) were trapped, including eight genera of Scolytidae, (*Xyleborus, Cryphalus, Polygraphs, Xyloterus, Ips, Dendroctonus, Orthotomicus* and *Scolytus*), totaling 16 species. The present condition of bark beetle quarantine in China is briefly discussed, and the broad research trends are outlined.

## Introduction

### Status of bark beetle quarantine of China and research trends abroad

The quantity of imported timber has increased rapidly with the constant economic development of China. Since 1999, the quantity of imported timber is over × 10^7^m^3^ annually and around 1.3∼1.4 × 10^7^m^3^ in recent years. Longicorn beetles, bark beetles and termites are important timber pests that might be present in imported timber and introduced to China, which could cause considerable damage to Chinese forestry.

Many species of bark beetles were intercepted from imported timber by the State Administration of Supervision, Inspection and Quarantine of the People's Republic of China in recent years. It was reported that 23 species were intercepted from imported timber and wooden packing from 1993 to 1998, in which were categorized to genera including *Xyloterus, Hylurgus, Ips, Scolytoplatypus, Hypothenemus* etc (Guan and Sun, 2001). Especially in recent years, the outbreak of the red turpentine beetle, *Dendroctonus valens*, which spread over China through timber import, occurred in Shanxi, Hebei and Henan Provinces in 1999. At the beginning of 2000, the total area affected was 52580hm^2^ in these three provinces, and more than 6 million Chinese pines, *Pinus tabulaeformis*, died. In 2001, the damage of this pest was found again in Yan'an city, Shanxi Province, spreading rapidly (Yan et al., 2003). Therefore, it is urgent to strengthen future quarantine efforts to prevent bark beetles, such as the red turpentine beetle, from causing widespread damage in China.

At present in China, the main method of bark beetle quarantine is to check the cargo by hand. It is hard to avoid oversight by only spot-checking the large amount of timber 2 to 3 times, when the container is full of cargo. The effect of quarantine is considerably limited by lack of advanced equipment and methods. New technology for quarantine is therefore needed (Yang, 1993).

In North America and Europe, much research has been done on bark beetle host orientation (Birch et al., 1980), interspecific olfactory communications, interspecific competition (Byers and Wood, 1980), the effect of conifer oleoresin released during bark beetle attack (Birgersson and Bergström, 1989), the effects of host volatiles on bark beetle's reaction to pheromone (Byers, 1992) and integrated management (Evans and Fielding, 1994). In particular, the organs which produce pheromone, and the methods of pheromone extraction and isolation, have been successfully studied, and the components and structures of the pheromones have been identified (Bell and Carde, 1990). Since 1995, research on the application of bark beetle semiochemicals in monitoring the dynamic of exotic species of bark beetles has been carried out in Canada (Leland, 2002). There is no report of applying bark beetle semiochemicals for quarantine in China.

### Characteristics of bark beetle semiochemicals

Semiochemicals are chemicals that mediate interactions between organisms. Semiochemicals are subdivided into kairomones and pheromones depending on whether the interactions are interspecific or intraspecific, respectively. Semiochemicals which are used in intraspecific communication are referred to as pheromones. Behavioral responses to pheromones include searching for mates by one sex (e.g., sex pheromones), aggregation of both sexes at a host plant (e.g., attractant or aggregation pheromones), and dispersal of both sexes away from a specific area (e.g., inhibitor or antiaggregation pheromones). Semiochemicals used in interspecific communication are referred to as kairomones when the species receiving the chemical message benefits and allomones when the emitter of the chemical message benefits at the expense of the receiver (Skillen et al., 1997). Semiochemicals from both trees and bark beetles influence many behavioral actions of a bark beetle during its life cycle (Byers, 1995).

Monoterpenoids (such as α-pinene, myrcene, terpinolene, β-pinene) and turpentine play an important role in bark beetle host selection, while the ethanol released by of decaying wood tissues that are infected by microorganisms is also used in host search. These compounds are synergic. Generally speaking, monoterpenoids and turpentine released by the host, or the mixture of these two components, are highly attractive to the bark beetle. Bark beetles can discover damaged trees by a low monoterpenoid release rate and a medium ethanol release rate by the host. If the host's resistance is not strong, “pioneer” bark beetles chew on the tree and stimulate it to release monoterpenoids that attract other bark beetles to aggregate, which results in a mass attack. If the host releases high amounts of ethanol, it indicates that the host has decayed and is inadequate for the survival and propagation of bark beetles (Klimetzek and Köller, 1986).

When “pioneers” bite into the bark, if the host is found to be suitable, they begin releasing the primary aggregation pheromone that attracts other adults of the same species. The male of multi-mate species (such as *Ips*) attacks the host first, and then the females find the gallery entrance mined by the male and enter it. After the mating, the females mine into the inner bark tissue and oviposit. However, the female of single-mate species (such as *Scolytus*) act as the “pioneers”. In these two instances, after more bark beetles come, they begin to release aggregation pheromone to attract more and more bark beetles, until the whole tree is mass colonized.

The pheromone secreted by bark beetles mainly exists in the feces of males. The pheromone of most species is secreted by the alimentary canal, especially the hindgut, and terpenols are the main components (such as ipsenol, ipsdienol, cis-verbenol, trans-verbenol, verbenone), which function in aggregation. However, it was found that verbenone can inhibit the reaction of bark beetles to their pheromones at certain concentrations (Bell and Carde, 1990). On the other hand, damaged trees will release some compounds that attract bark beetles and are used by bark beetles to synthesize pheromones (Bell and Carde, 1990). Species-specific characteristics of the semiochemicals are not distinct.

Some species can utilize interspecific competition to discover adequate hosts by the compounds released during establishment by other species. These compounds are the kairomones of the host or the pheromones of other species (Birch et al., 1980). Many species of bark beetles may exist in the same environment, and they will compete for one resource. The semiochemicals released into the environment by any species may be received by other species that then stimulate aggregation to the same resource (Bell and Carde, 1990).

The research discussed above has established a basis for the development of bark beetle aggregation and application for use in quarantine.

## Methods and Materials

### Study Area

The trap trial was carried out in imported timber in Zhangjiagang Port (where timber is imported from South-East Asia, Africa and Oceania) and Taicang Port (where timber is imported from Russia).

### Experimental Reagent

Two isomeric compounds of α-pinene, ethanol, trans-verbenol, verbenone, Camphene and Isononylaldehyde were used in these experiments. These reagents were provided by Aldrich (www.sigmaaldrich.com), and they were all analytical reagents.

### Experiment of attractive components selection

The attractant was injected into the core of Lindgren funnelform trap (www.pherotech.com/lindgren_funnel_trap.html), that were then hung at random every 10 meters among the imported timber on a ship. Each comparison of various lure blends trapped had 10 replications per treatment, and in every treatment, a control trap was set without any attractant. A very large number of traps were used during the experiment. 12 hours later, the bark beetles trapped in every trap were collected, and the numbers of bark beetle trapped was recorded. The percentage trapped by each attractant was calculated relative to total number trapped for each group. Variance analysis and SSR test were done by SAS software. The trapped bark beetles were dried for species identification. After species identification, the relative species-level responses to each attractant between 2 study sites were evaluated separately.

**Table 1.**
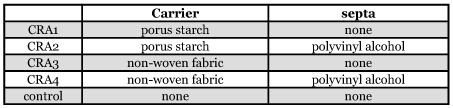
Carrier and septa of controlling released attractants (CRA)

### Experiment of controlling release function of attractant

Four controlled released attractants (Table 1) were tested. Formulated lures were placed in the trap core. The bark beetles trapped was determined every 8 hours, until no bark beetles were caught. Bark beetles trapped were released. The length of time that the lures remained active was noted. In the controlled release experiment, for each treatment the dosage of semiochemicals absorbed by the carrier was 5 ml. The controlling released attractants were prepared by National Engineering Research Center for Dyeing and Finishing of Textiles of China.

**Table 3.**
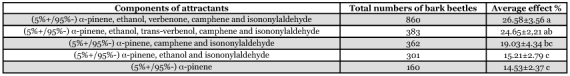
*SSR* test of the relative effect of attractants of (5%+/95%-) a-pinene added minor

### Species identification of trapped bark beetles

Dry specimens of trapped bark beetles were sent to Institute of Zoology, Chinese Academy of Sciences for species identification.

## Results

### Experiment of attractive components selection

The result of the experiment of different ratios of two isomeric compounds of α-pinene in the attractant showed that the attraction to (5%+/95%-) α-pinene was higher than for other compounds (Table 2). A significant treatment effect was observed (F_5,54_=21.97>F_0.05_=2.39, P=6.13E-12).

**Table 2.**
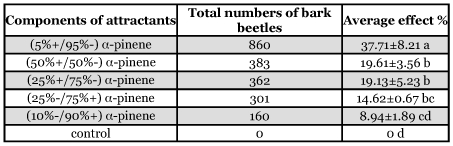
*SSR* test of the relative effect of attractants of two isomeric compounds of a-pinene

**Table 4.**
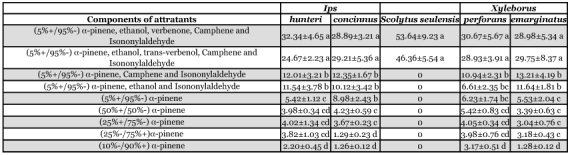
*SSR* test of the relative species-level responses to each attractant in Zhangjiagang Port

After adding the minor components α-pinene, ethanol, verbenone, camphene and isononylaldehyde to (5%+/95%-) α -pinene, attraction was higher than for the other compounds, except for the attractant in which trans-verbenol was substituted for verbeone (Table 3). A significant treatment effect was observed (F_5,54_=7.54>F_0.05_=2.58, P=9.73E-05).

Comparing the response of different species to the attractants, the attractant containing (5%+/95%-) α -pinene, ethanol, verbenone, camphene and isononylaldehyde and the one containing (5%+/95%-) α-pinene, ethanol, trans-verbenol, camphene and isononylaldehyde attracted more bark beetle species than other attractants, including some damaging species, such as *Dendroctonus, Scolytus* and *Ips* (Tables 4, 5).

### Experiment of controlling release function of attractant

**Figure 1:**
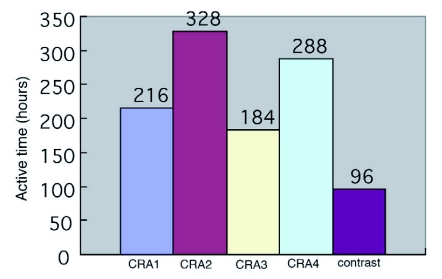
Active time of controlled released attractants (CRA). Numbers above bars represent the numbers of beetles trapped.

Figure 1 shows that the active time of controlled released attractants using the porous starch and septa of polyvinyl alcohol as carriers was longer than the other carriers.

### Species identification of trapped bark beetles

Insects of two subfamilies (Scolytinae and Platypodinae) of Curculionidae were trapped, including eight genera of Scolytinae, (*Xyleborus, Cryphalus, Polygraphs, Xyloterus, Ips, Dendroctonus, Orthotomicus* and *Scolytus*), totaling 16 species (Table 6).

**Table 5.**
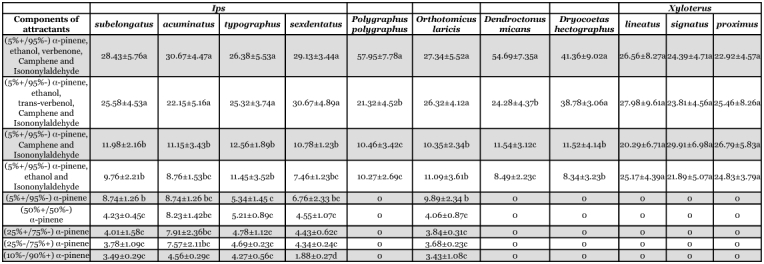
*SSR* test of the relative species-level responses to each attractant in Taicang Port

## Discussion

Pheromones have been used in pest forecast and control for years, and field trials have shown that it is efficient in many countries. But there is still no report of applying bark beetle semiochemicals as a quarantine method in China.

Yang (1993) suggested the use of insect attractants for plant quarantine, and discussed how they could be applied. In order to discover new methods of bark beetle quarantine in China and improve the quarantine effect, the General Administration of Quality Supervision, Inspection and Quarantine of the People's Republic of China has established the research subject titled “Application of Bark Beetle Aggregation Pheromone in Quarantine”, and cooperative research is done by Wuxi Entry-Exit Inspection and Quarantine and the College of Plant Protection, Nanjing Agricultural University. After three years of research, many attractants and controlled release attractants were prepared based on the attractive components of host and effective components of bark beetle semiochemicals.

**Table 6.**
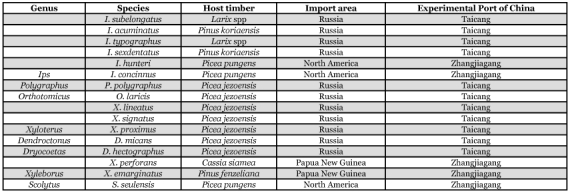
Species identification of trapped bark beetles

The results showed that the attractants are effective in trapping bark beetles in imported timber. Beetles from two subfamilies of Curculionidae were trapped, including eight genera in Scolytinae, totaling 16 species, including some species that damage trees, such as *Dendroctonus, Scolytus, Ips*. The result of the trapping trial showed that the attractants were efficient, and that the technique can be modified for timber quarantine in China. Research is continuing to develop new formulations for attractants and practical techniques for quarantine, so as to improve the technique and develop it for bark beetle quarantine in China.

## References

[bibr01] Bell WJ Carde RT 1995 *Chemical Ecology of Insects*2nd London, New York Chapman and Hall

[bibr02] Birch MC Svihra P Paine TD 1980 Influence of chemically mediated behavior on host tree colonization by 4 cohabiting species of bark beetles. *Journal of Chemical Ecology* 6 395 414

[bibr03] Birgersson G Bergström G 1989 Volatiles released from individual spruce bark beetle entrance holes: quantitative variations during the first week of attack. *Journal of Chemical Ecology* 15 2465 2484 10.1007/BF01020377 24271543

[bibr04] Byers JA 1992 Attraction of bark beetles, *Tomicus piniperda, Hylurgops palliatus*, and *Trypodendron domesticum* and other insects to short-chain alcohols and monoterpenes. *Journal of Chemical Ecology* 18 2385 2402 10.1007/BF00984957 24254878

[bibr05] Byers JA 1995 Host tree chemistry affecting colonization in. bark beetles. Cardé RT Bell WJ *Chemical ecology of insects*2nd 154 213 New York Chapman and Hall

[bibr06] Byers JA Wood DL 1980 Interspecific inhibition of the response of the bark beetles, *Dendroctonus brevicomis* and *Ips paraconfusus* , to their pheromones in the field. *Journal of Chemical Ecology* 6 149 164 10.1007/BF00988631 24420423

[bibr07] Evans HF Fielding NJ 1994 Integrated management of *Dendroctonus micans* in the forest. *Ecology and Management* 65 17 30

[bibr08] Guan XM Sun XG 2001 The advances in the research on wood bark beetles. *Forest Science and Technology* 23 8 10

[bibr09] Klimetzek D Köller J Vite JP 1986 Dosage response to ethanol mediates host selection by secondary bark beetles. *Naturwissenschaften* 73 270 272

[bibr10] Leland MH 2002 Alien Forest Pests: Canadian forest service research on a global problem. *Oral Report in Chinese Academy of Forestry* Canadian Forest Service Pacific Forestry Centre www.earthscape.org/r1/cfso1/cfso1.html

[bibr11] Skillen EL Berisford CW Camann MA Reardon RC 1997 *Semiochemicals of forest and shade tree insects in North America and management implications*. FHTET-96-15. U.S. Department of Agriculture, Forest Service Morgantown

[bibr12] Yan ZL Sun JH Zhang ZN 2003 An invasive alien species *Dendroctonus valens* : damage in forestry in China and its semiochemicals. *Entomological Knowledge* 40 399 404

[bibr13] Yang GH 1993 Imagine of application of insect attractant in plant quarantine. *Plant Quarantine* 7 226 229

